# The effect of the teacher-student relationship on the academic adjustment of returned migrant children: the chain mediating role of school attitude and resilience

**DOI:** 10.3389/fpsyg.2024.1464904

**Published:** 2025-01-03

**Authors:** Chuanyan Wang

**Affiliations:** The Institute of Education, China University of Geosciences, Wuhan, China

**Keywords:** returned migrant children, academic adjustment, teacher-student relationship, school attitude, resilience

## Abstract

**Introduction:**

Returned migrant children have not received widespread attention in China, and research on their academic adjustment is still limited. Teachers are important individuals who influence the academic development of returned migrant children, and the aim of the study is to analyze the mechanism by which the teacher-student relationship affects their academic adjustment.

**Methods:**

This study followed a sample of 1921 returned migrant children across 8 counties in central and western China, using an academic adjustment scale, a teacher-student relationship scale, a school attitude scale, and a resilience scale. Pearson-moment correlations were used to analyze the the correlations among all variables, and the PROCESS macro (Model 6) in SPSS was used to examine the mediating effects.

**Results:**

The findings indicated that the teacher-student relationship significantly and positively predicted academic adjustment. Furthermore, the relationship indirectly influenced academic adjustment through school attitude and resilience, which acted as chain mediators.

**Discussion:**

The findings highlight the importance of the teacher-student relationship in development outcomes. Moreover, early intervention and prevention efforts should be taken to improve their educational experience.

## 1 Introduction

In recent years, return migration has gained considerable attention as a more challenging process than simple reintegration into society (Markowitz and Stefansson, 2004). King (2000) has defined return migration as the process whereby individuals return to their country or place of origin after spending a considerable amount of time in another country or region. For returned migrant children, “return” means a new migration experience, and their “return” is atypical, as they are relocating to a place that may never have been home to them (Knörr, 2005; King et al., 2014). Earlier studies suggested that, due to cultural differences, returned migrant children often face academic challenges as well as psychological issues (Ní Laoire, 2011; Vathi et al., 2016).

Returned migrant children in China face unique challenges. Due to factors such as the depopulation policies of mega-cities and restrictions on post-compulsory education in inflow places, they often lack the opportunity or academic competitiveness to attend regular high schools and take college entrance exams. As a result, many migrant children are compelled to return to their hometowns for post-compulsory education (Ling, 2017). The “New Citizen Program” reported that, 922,000 migrant children returned to their hometowns in 2021 (Wei, 2023). Previous studies have demonstrated that returned migrant children in China encounter academic adjustment difficulties due to disparities in educational systems, school settings, teaching styles and content between the inflow areas and their hometowns, which leads to a decline in academic performance (Liu and Zhu, 2011; Koo et al., 2014; Ling, 2017).

Academic adjustment is generally described as the extent to which a student successfully addresses various educational demands and difficulties (Baker et al., 1985). Teachers, as “significant others” in students’ lives, play a crucial role in helping returned migrant children enhance their academic adaptability, and a positive teacher-student relationship is key to fulfilling this role. However, existing research has shown that teachers often perceive returned migrant children as low achievers, troublemakers, undisciplined, rude, and lacking identity (Hazzichristou and Hopf, 1995). In turn, returned migrant children and their parents also view teachers as more authoritarian, with some making offensive remarks and showing little respect for the diversity of their students (Vathi et al., 2016). This discordant relationship clearly impacts their academic adjustment. School attitude, as a potential predictor of the impact of teacher-student relationships on academic adjustment (Roeser and Midgley, 1996), and may operate through psychological resilience, an internal factor (Sanders et al., 2016). This study aims to explore the associations among teacher-student relationship, school attitude, resilience and academic adjustment, and further investigate their underlying mechanisms. The findings can help identify effective strategies to improve the academic adjustment of returned migrant children.

### 1.1 Association between teacher-student relationship and academic adjustment

The Coleman Report highlighted teachers as one of the most influential school factors affecting students’ academic performance (Coleman et al., 1966). Pianta (1999) has described a positive teacher-student relationship as open communication, emotional support, and academic guidance. He assessed the quality of the relationship through dimensions of closeness, conflict, and dependence. When the teacher-student relationship is marked by closeness, children tend to exhibit higher levels of overall academic adjustment compared to peers with lower levels of closeness (Birch and Ladd, 1997). Negative relational styles, characterized by high levels of conflict and dependence, are consistently associated with academic adjustment problems and play a significant role in predicting future school outcomes (Harmre and Pianta, 2011). Granot (2014) found that children with secure teacher-student attachments demonstrated fewer behavior problems, greater frustration tolerance, and better academic performance. Teachers also impact students’ long-run academic outcomes by fostering relationships that endure over time and influence broader aspects of their social capital, aspirations and life choices (Kraft et al., 2023).

From the students’ perspective, students who feel that their efforts are recognized by their teachers are more motivated to explore and learn, exhibit higher self-esteem and confidence in their academic abilities, are more receptive to instruction and criticism, and cope better with challenges (Roorda et al., 2011). Certain personality traits, such as motivational beliefs, values, and goals, are shaped and internalized through social forces, including teacher-student relationship in the classroom. These internalized resources help students adjust their academic behaviors and actively engage in the classroom, thereby enhancing academic adjustment (Zee et al., 2013). From the teachers’ perspective, positive relationships with students can motivate them to invest extra time and energy into promoting students’ success, while negative relationships are often linked to efforts to exclude students from the classroom (Pianta et al., 1995). Vathi (2015) pointed out in his research on Albania that teachers are crucial to returned migrant children’s progress in school, and a positive relationship with teachers can provide them with greater academic support.

Based on the above review, we propose Hypothesis H1: The teacher-student relationship is positively associated with the academic adjustment of returned migrant children.

### 1.2 Association between teacher-student relationship, school attitude, and academic adjustment

School attitude refers to students’ evaluations and behavioral tendencies toward school life, encompassing both school liking and school avoidance (Ladd, 1990). Teachers’ perceptions of support, satisfaction, and conflict significantly predict students’ attitudes toward school (Huan et al., 2012). When the teacher-student relationship is positive, students tend to be more actively involved in school activities, leading to a greater liking for school. In contrast, students would be school avoidant (Martin and Collie, 2018). Conflict in the teacher-student relationship is positively associated with school avoidance and negatively related to school liking, self-regulation, and cooperation in the classroom (Birch and Ladd, 1997).

Previous research has demonstrated that students’ school attitudes are closely linked to their academic adjustment (Filippello et al., 2020). A strong sense of belonging to school serves as a protective factor against learned helplessness, whereas a sense of school rejection acts as a risk factor, triggering stress and hindering academic adjustment (Arslan, 2016; Raufelder and Kulakow, 2022). Without an appropriate attitude toward school, students may struggle to adapt to changing environments and acquire the new knowledge and skills necessary for academic success (Raabe, 2019). In turn, highly positive school attitudes can boost students’ internal motivation and interest in learning, ultimately leading to better academic performance (Kpolovie et al., 2014). Szydłowska et al. (2019) found that returned migrant children in Polish experiencing critical comments from teachers, who appeared unaware of their challenges, may adopt a passive attitude and struggle with low self-esteem, which could further complicate their integration into new environments.

Based on these findings, we propose Hypothesis H2: The effect of the teacher-student relationship on returned migrant children’s academic adjustment is mediated by their school attitudes.

### 1.3 Association between teacher-student relationship, resilience, and academic adjustment

Resilience is the process, ability, or outcome of adapting well despite difficult or threatening situations (Masten et al., 1990). Resilience theory posits that children experiencing even extreme adversity can achieve positive outcomes through various resources that buffer or mitigate stressors (Masten, 2014). However, children cannot achieve resilience on their own, and teachers are a significant resource for promoting resilience in at-risk children (Neville et al., 2019). Attachment research has proved teachers to be a secure base for high-risk children (Pianta et al., 1995). A constructive, trustful, and supportive teacher-student relationship is the foundation for resilient development, and high-risk children are likely to become or remain resilient to adversity as long as they have reliable teacher-student relationships (Diers, 2020). A longitudinal study also revealed that children and adolescents with high resilience identify teachers as their main attachment figures outside the family (Werner, 2006).

In general, individuals with higher resilience are better equipped to adapt to changing or stressful environments, adjust their behaviors as needed, employ problem-solving strategies flexibly, and recover from traumatic experiences (Block and Block, 2006). Research concerning high school students showed a moderate, positive relationship between resilience and active coping (Ahin and Hepsütlü, 2018). Vathi et al. (2016) reported that discriminatory reactions from teachers appears as an important factor upon returned migrant children’s ability to adapt to a new environment. A study of Latino migrant children revealed that resilience was positively associated with academic efficacy and negatively associated with academic adjustment problems (Taylor et al., 2019).

Based on these studies, we propose Hypothesis H3: The effect of the teacher-student relationship on returned migrant children’s academic adjustment is mediated by their resilience.

### 1.4 Association between school attitude and resilience

Schools not only serve as protective environments for children in need, but also play a vital role in their social and academic development. Students who perceive positive teacher-student relationships in school—characterized by respect, support, and care—tend to report a stronger sense of school belonging. Positive attitudes toward school enhance their academic self-efficacy and self-consciousness (Roeser and Midgley, 1996). School attitudes are shaped by a complex interplay of cognitive, emotional, and social factors and are closely related to the psychological environment (Moè et al., 2009).

Positive attitudes toward school improve children’s emotional regulation, self-confidence and the development of a broader support network (Wright, 2013). Sun and Wu (2011) have found that middle school students’ school attitudes significantly and positively predict resilience. The more favorable the school climate students perceive, the easier it is for them to develop psychologically resilient traits that build their internal resources, such as a strong sense of purpose and a positive outlook on life (Li et al., 2022). Students with positive school attitudes are more likely to exhibit resilient behavior in communication, self-esteem, help-seeking, goal-setting and aspirations, and are better equipped to cope with academic challenges (Stewart and Sun, 2004). In addition, educational activities in schools do not occur in isolation, and various elements of the school settings influence students’ social and emotional development, playing a crucial role in fostering resilience (Wright, 2013). Thus, school attitude serves as a protective factor for resilience.

On the base of the above review, we propose Hypothesis H4: School attitude is positively associated with resilience, and both factors acted as chain mediators between teacher-student relationship and returned migrant children’s academic adjustment.

### 1.5 Current study

**Figure 1:**
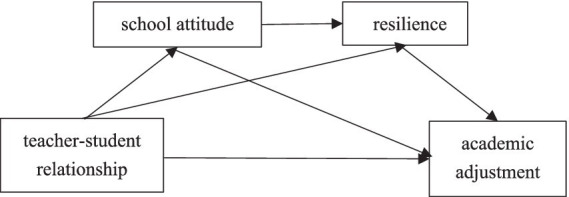
The hypothesized model of the teacher-student relationship associated with academic adjustment.

Returned migrant children have received limited attention in China, and the impact of the teacher-student relationship on their academic adjustment has not been widely researched, as reflected in the scarcity of published research on this topic. The purpose of this study is to examine how the teacher-student relationship influences returned migrant children’s school adjustment, with a focus on the roles of school attitude and resilience in a chain mediating model. We specifically focus on returned migrant children in middle and high schools in China. On the basis of the review above, we hypothesize that the teacher-student relationship is significantly associated with returned migrant children’s academic adjustment and exerts an indirect effect through school attitude and resilience, with these two factors acting as chain mediators in the mechanism (see Figure 1).

## 2 Methods

### 2.1 Participants

The participants were returned migrant children from 32 middle schools and high schools across eight counties in four provinces in central and western China in 2023. After reading and signing the informed consent form, the participants completed the questionnaire. A total of 1988 returned migrant children voluntarily participated in the study, with 67 protocols excluded due to missing or invalid responses, leaving 1,912 valid data. The sample included 48.2% boys, 55.5% middle school students, and 45.1% born in the inflow areas. The majority of participants were inter-provincial migrants (75.8%), with 61.3% having returned to rural schools in their hometowns. The average of age at return was 11 years, and the average duration of return was 3.9 years.

### 2.2 Procedure

Data were collected between March and September 2023, following approval from the local education authorities’ ethics committee. We selected eight counties in four central and western provinces as the survey areas. The migration trend in China typically flows from economically underdeveloped central and western provinces to more economically developed eastern provinces. Consequently, returned migrant children are predominantly found in central and western provinces (Koo et al., 2014). The cities under study vary in terms of location and economic conditions, collectively reflecting the diverse circumstances of returned migrant children. The local education authority recommended four middle and high schools for participation: one middle school and one high school from a township, and the other two from urban areas. This proposal was based on the distribution of returned migrant children in the region. In each school, participants were recruited following the principle of cluster sampling. To ensure the authenticity and reliability of the questionnaire, the respondents were guided by trained research assistants when the questionnaire was completed (30–40 min). The participants were assured that their responses would remain anonymous and that they could withdraw from the study at any time. As a token of appreciation, each child received a small gift upon completing the survey.

### 2.3 Measures

#### 2.3.1 Academic adjustment

Academic adjustment was student-reported using items from the academic adjustment sub-scale of the School Life Adjustment Scale for children of migrant workers (Xu, 2010). The scale consists of 12 items, including three dimensions: adjustment to the learning environment, adjustment to the learning methodology and academic outcomes. The items were rated on a 5-point Likert scale (1 = not at all, 5 = very much so). The items related to “adjustment to the learning environment” were scored in the reverse, meaning a response of “1” on item like “I do not fit in here” was scored as “5” in the final aggregation. A higher total score indicates a higher level of academic adjustment. In this study, the Cronbach’s alpha coefficient of the scale was 0.836.

#### 2.3.2 Teacher-student relationship

Teacher-student relationship was student-reported via four items from the teacher-student relationship sub-scale of the School Adjustment Scale (Cui, 2008). Students rated items such as “I try to avoid contact with teachers” and “1 do not think my teachers understand me” on a five 5-point Likert scale (1 = not at all, 5 = very much so). All the items were reverse-scored and then converted to positive scores in the final aggregation. A higher score indicates a better teacher-student relationship. The Cronbach’s alpha coefficient of the scale in this study was 0.799.

#### 2.3.3 School attitude

School attitude was student-reported using 4 items from the school attitude sub-scale of the School Adjustment Scale (Cui, 2008). The items included: “I am comfortable at school,” “I hate going to school” “1 am depressed at school” and “I am satisfied with my school life.” The scale was rated on a 5-point Likert scale ranging from 1 (not at all) to 5 (very much so). The reverse-scored items were converted to positive scores when calculating the total score. A higher score indicated more positive school attitudes. The Cronbach’s alpha coefficient for the scale in this study was 0.787.

#### 2.3.4 Resilience

The Chinese version of the Connor-Davidson Resilience Scale (CD-RISC, Connor and Davidson, 2003) was used, revised by Yu and Zhang (2007). The 25-item questionnaire included items such as “1 have a strong sense of purpose in life,” and “Good or bad, I believe that most things happen for a reason.” The scale covers psychological characteristics that are relevant to the Chinese population (Yu and Zhang, 2007) and have been widely used in research across various groups in China. Items were rated on a 5-point scale ranging from 1 (not at all) to 5 (true nearly all the time). Higher scores represented higher levels of resilience. The Cronbach’s alpha coefficient for this scale in this study was 0.929.

#### 2.3.5 Control variables

Previous research has shown that returned migrant children’s academic adjustment may be influenced by their age (Hazzichristou and Hopf, 1995), family structure (Ní Laoire, 2011) and family relations (King et al., 2014). Thus, we measured age, family structure and family relations as control variables in the current study.

### 2.4 Data analyses

Descriptive and correlational statistics were first calculated for all the variables. Pearson product–moment correlations were used to assess the strength and direction of the linear relationships between pairs of variables. Next, we analyzed the influence of the teacher-student relationship on returned migrant children’s academic adjustment and investigated the chain mediating roles of school attitude and resilience. In accordance with Hayes (2013), PROCESS programmed Model 6 to estimate the indirect effect of the independent variable (X) on the dependent variable (Y) through two mediators (M1 and M2). The SPSS macro program PROCESS was used for testing the proposed mediating effects.

## 3 Results

### 3.1 Preliminary analysis

Table 1 presented the means and standard deviations of the variables. The academic adjustment, teacher-student relationship, school attitudes, and resilience of the returned migrant children were found to be at moderate levels. The survey revealed significant variability among individuals. Some returnees expressed dissatisfaction with schools in their hometowns, citing the superior school environments they experienced in inflow cities. In contrast, other returnees reported a strong attachment to their hometowns schools, having resided there at an early age. This variability likely explains why the mean values of these variables were close to moderate.

**Table 1:** Descriptive statistics and correlations between all variables.

	*M*	SD	1	2	3	4	5	6
Age	14.94	1.787	–					
Family structure	1.92	1.037	−0.107^**^	–				
Family relations	3.95	0.925	−0.074^**^	−0.056^*^	–			
Academic adjustment	3.49	0.700	−0.171^**^	−0.052^*^	0.228^**^	–		
Teacher-student relationship	3.78	0.977	−0.094^**^	−0.046^*^	0.171^**^	0.498^**^	–	
School attitude	3.43	1.010	−0.185^**^	−0.002	0.217^**^	0.538^**^	0.502^**^	–
Resilience	3.28	0.735	−0.075^**^	−0.080^**^	0.246^**^	0.614^**^	0.373^**^	0.450^**^

Family structure: 1 = nuclear family, 2 = stem family, 3 = single-parent family, 4 = combined family, 5 = other; Family relations: 1 = very bad, 2 = not so good, 3 = average, 4 = better, 5 = very good; ^*^p < 0.05, ^**^p < 0.01, ^***^p < 0.001.

The correlations between all the variables were calculated using Pearson’s product correlation coefficient (see Table 1). The results revealed that the teacher-student relationship was significantly positively associated with school attitude (*r* = 0.502, *p* < 0.01), resilience (*r* = 0.373, *p* < 0.01), and academic adjustment (*r* = 0.498, *p* < 0.01). Furthermore, school attitude was significantly positively associated with resilience (*r* = 0.450, *p* < 0.01), and resilience was significantly positively associated with academic adjustment (*r* = 0.614, *p* < 0.01). All four primary variables were positively correlated and reached statistical significance, which provided preliminary evidence for the hypotheses.

Furthermore, the results showed that individual characteristic variables, such as age and family structure were negatively correlated with the primary variables, while family relations showed a positive correlation with the primary variables. Thus, these variables were included as control variables in the subsequent path analysis.

Harman’s single-factor test was conducted to examine the presence of common method bias. Seven common factors with eigenvalues greater than 1 were extracted, with the first factor explaining 30.79% of the variance, which was below 40% threshold. This suggests that common method bias is not a significant issue in this study.

### 3.2 Multiple mediation effect analysis

Using the PROCESS 4.1 plug-in in SPSS, the direct and indirect effects of the teacher-student relationship on academic adjustment were tested (see Figure 1). A bias-corrected non-parametric percentile bootstrap method was applied, with 5,000 repetitions and a 95% confidence intervals. In this analysis, the teacher-student relationship was the independent variable, academic adjustment was the dependent variable, and school attitude and resilience served as the mediating variables. The analysis controlled for gender, family structure, and family relations as covariates (see Table 2).

**Table 2:** Results of multiple mediation model analysis.

Regression equation		Fit index			Regression coefficients and significance	
Outcome variables	Predictor variables	*R*	*R* ^2^	*F*	*β*	*t*
Academic adjustment	Age	0.529	0.280	149.090^***^	−0.136	−6.172^***^
	Family structure				−0.062	−2.842^**^
	Family relations				0.138	6.240^***^
	Teacher-student relationship				0.439	20.287^***^
School attitude	Age	0.532	0.283	150.852^***^	−0.132	−6.026^***^
	Family structure				0.002	0.918
	Family relations				0.128	5.803^***^
	Teacher-student relationship				0.462	20.929^***^
Resilience	Age	0.515	0.265	110.326^***^	−0.003	−0.116
	Family structure				−0.075	−3.390^**^
	Family relations				0.141	6.279^***^
	Teacher-student relationship				0.168	6.604^***^
	School attitude				0.348	13.452^***^
Academic adjustment	Age	0.719	0.504	259.303^***^	−0.086	−4.659^***^
	Family structure				−0.032	−1.744
	Family relations				0.032	1.696
	Teacher-student relationship				0.209	9.908^***^
	School attitude				0.223	9.904^***^
	Resilience				0.416	19.811^***^

^*^p < 0.05, ^**^p < 0.01, ^***^p < 0.001.

**Figure 2:**
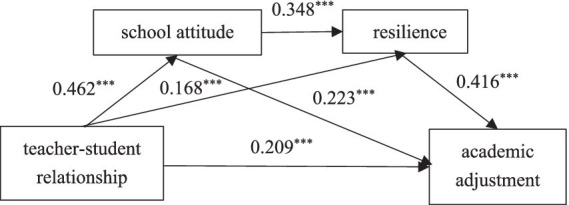
The chain mediating model.

Cook’s distance was used to assess potential outliers in the regression models. The results showed that the maximum Cook’s distance values for the four models were 0.020, 0.014, 0.018 and 0.023 respectively, all of which were smaller than the critical value of “1.” This indicated that there were no influential outliers in the models, suggesting the reliability of the regression analyses (Figure 2).

First, in the direct effect model, where the independent variable (teacher-student relationship) was correlated with the dependent variable (academic adjustment), and after controlling for demographic variables, the teacher-student relationship significantly and positively predicted academic adjustment (*β* = 0.439, *p* < 0.001). Second, in the model examining the relationships between the independent variable and the mediating variables, with the inclusion of control variables, the teacher-student relationship significantly and positively predicted school attitude (*β* = 0.462, *p* < 0.001) and resilience (*β* = 0.168, *p* < 0.001), and school attitude was positively associated with resilience (*β* = 0.348, *p* < 0.001). Third, in the indirect effect model, where both the independent variable and mediating variables (school attitude and resilience) were correlated with the dependent variable, with control variables included, both school attitude (*β* = 0.223, *p* < 0.001) and resilience (*β* = 0.416, *p* < 0.001) significantly and positively predicted academic adjustment. Even in this model, the teacher-student relationship remained a significant predictor of academic adjustment (*β* = 0.209, *p* < 0.001). These results suggested school attitude and resilience play a significant chain-mediating role in the relationship between the teacher-student relationship and academic adjustment (Figure 2).

The results of the mediating effect test showed that (see Table 3), the value of the direct effect of the teacher-student relationship on academic adjustment was 0.152, accounting for 46.8% of the total effect, thus confirming Hypothesis H1. Additionally, the teacher-student relationship influenced academic adjustment through the chain mediation of school attitude and resilience, and the total mediating effect was 0.173, accounting for 53.2% of the total effect. Specifically, the mediating effect was divided into three paths: indirect effect through the teacher-student relationship → school attitude → academic adjustment (0.075), and research Hypothesis H2 was verified; indirect effect through the teacher-student relationship → resilience → academic adjustment (0.050), confirming Hypothesis H3; indirect effect through teacher-student relationship → school attitude → resilience → academic adjustment (0.048), which supported Hypothesis H4. The 95% confidence intervals of the mediation effects for all the above paths did not contain “0,” indicating that all the mediation effects were statistically significant.

**Table 3:** Effect values and 95% confidence intervals for each path.

	Intermediary path	Effect value	Boot SE	95% CI	Effect percentage
Direct effect	Teacher-student relationship → academic adjustment	0.152	0.153	[0.122, 0.182]	46.8%
Indirect effect	Total indirect effect	0.173	0.014	[0.148, 0.200]	53.2%
	Teacher-student relationship → school attitude → academic adjustment	0.075	0.009	[0.057, 0.093]	23%
	Teacher-student relationship → resilience → academic adjustment	0.050	0.010	[0.032, 0.071]	15.4%
	Teacher-student relationship → school attitude → resilience → academic adjustment	0.048	0.006	[0.038, 0.060]	14.8%
Total effect		0.325	0.016	[0.294, 0.357]	

## 4 Discussion

The results suggested that the teacher-student relationship had a direct effect on returned migrant children’s academic adjustment. Ryan and Patrick (2001) indicated that the quality of the teacher-student relationship promotes and shapes students’ academic expectations by influencing their learning goals, engagement, interest in learning, and values, as well as attributions to sources of success or failure. When students perceive that teachers promote communication and mutual respect, their motivation to learn changes positively, favoring a more adaptive mode of learning. Upon returning to their hometowns, children encounter numerous challenges in adapting to their new school, such as adjusting to differences in curriculum, teaching styles, and scheduling. Additionally, these children often bring with them habits developed in inflow areas, such as speaking Mandarin, which can be different from the practices in their hometowns. In this context, a harmonious teacher-student relationship is crucial in helping them overcome challenges and adapt to their new educational environment.

As hypothesized, the study revealed that school attitude partially mediated the effect of the teacher-student relationship on returned migrant children’s academic adjustment. A positive teacher-student relationship, characterized by perceived teacher support, is a strong predictor of students’ increased interest in learning and improved school behavior (Woolley and Bowen, 2007). Murdock (1999) identified teachers’ support and expectations as the strongest and most consistent predictors of students’ school engagement and compliance in his study of school alienation. Similarly, Wan et al. (2010) found that conflicting student-teacher relationships have a detrimental effect on students’ attitudes toward school, fostering an environment of disengagement. In this study, returned migrant children who reported positive relationships with their teachers felt supported both academically and personally by their teachers. This support helped them integrate quickly into their schools and make progress in their studies. On the other hand, returned migrant children with negative teacher-student relationships often felt that teachers held prejudices against them, perceiving them as problematic students who returned due to poor academic performance. These children received little additional support and were sometimes treated unfairly, leading to negative perceptions of their new schools and exacerbating their sense of maladjustment. These findings underscore the significant role teachers play in shaping returned migrant children’s school attitudes, which in turn affect their academic adjustment.

Resilience also played a partially mediating role in the relationship between the teacher-student relationship and returned migrant children’s academic adjustment. Positive interpersonal relationships at school are considered a critical resilience resource for high-risk adolescents (Noltemeyer and Bush, 2013). Resilience acts as a protective factor for academic adjustment by help individuals adopt positive thinking, problem-focused coping strategies, and a proactive approach to seeking support. These traits enable them to better adapt to changes in the learning environment and achieve academic success (Meneghel et al., 2019). The current research revealed that some respondents had complex migration experiences, initially being left behind in their hometowns, later joining their parents in the cities, and eventually returning to their hometowns alone. This series of migration-related challenges exposed them to multiple cumulative risks. In such cases, positive teacher-student relationships can serve as a key internal resource, strengthening resilience and helping returned migrant children navigate these difficulties. Returned migrant children with high resilience tend to activate their internal motivation, adopt effective learning strategies, and seek help from others when needed. For instance, some participants described how they coped with academic adjustment barriers using phrases like “make the first move,” “self-planning,” “put myself in one’s shoes” and “figure it out.” These expressions indicate a proactive, solution-focused approach to overcoming challenges, highlighting how resilience, fostered through positive teacher-student relationships, can drive academic adjustment.

The study also revealed that school attitude and resilience act as chain mediators of the effect of the teacher-student relationship on the academic adjustment of returned migrant children. This finding highlights that a positive school attitude can improve children’s resilience, and that the process of academic adjustment is influenced by a multitude of factors, both external to the school and internal to the individual. A longitudinal study by Read et al. (2015) found that a school’s intentional efforts to make students feel welcomed, connected, and belonged through a cross-cultural approach significantly improved students’ perceptions of the school atmosphere, as well as their resilience and wellbeing 18 months later. Castro-Olivo et al. (2013) also emphasized the importance of school belonging as a resource for resilience, suggesting that when students feel a sense of school belonging, they are less likely to experience maladjustment or engage in risky behaviors, and they tend to demonstrate better emotional self-regulation and academic achievement. For returned migrant children, establishing positive interpersonal relationships at school increases their satisfaction with the school environment, which in turn helps them adjust their mindset and focus on their academic pursuits.

In addition, control variables such as age, family structure, and family relations also play significant roles in the academic adjustment of returned migrant children. Hazzichristou and Hopf (1995) found that older returned migrant children tend to face greater challenges in school, with those who return after the age of nine having a much lower likelihood of academic success. Similarly, Kunuroglu et al. (2018) emphasized that migrant parents place great importance on the critical age for their children’s return. While perceptions of this “critical age” vary, there is a general consensus that children should return before puberty to have sufficient time to adjust to the educational environment. For returned migrant children in China, the older they return, the greater the academic discrepancies they encounter in terms of curriculum and teaching methods. Furthermore, children from divorced families or those with poor relationships with their parents or guardians, they may struggle to receive the necessary family support to develop their resilience or positive school attitudes, further hindering their academic adjustment.

### 4.1 Limitations

One of the most notable limitations of this study was the absence of repeated measures of the main variables. As time progresses, factors such as the teacher-student relationship, school attitude, resilience and academic adjustment are likely to change. Future research could benefit from repeated measures or longitudinal studies. Second, the cultural context of resilience measurement may introduce variability. Since resilience is influenced by cultural factors, it would be beneficial for future research to develop a resilience scale specifically designed for Chinese students to better capture cultural nuances and provide more accurate insights into resilience in this population. Third, the utilization of a self-reported survey methodology may introduce a degree of bias due to social desirability. The bias could be reduced by using observational measures or teacher-reported or peer-reported data. Additionally, it is necessary to further explore the mechanism through which the teacher-student relationship affect academic adjustment. Previous research has demonstrated that students with greater teacher-student relationships display better emotional and behavioral engagement in school and develop a sense of control (Skinner et al., 1998; Zimmer-Gembeck et al., 2006). Future studies could further explore the mediating roles of emotional engagement, behavioral regulation, and perceived control in the associations between the teacher-student relationship and academic adjustment.

### 4.2 Implication

The findings of this study have important implications for early intervention efforts. To improve the educational experiences of returned migrant children, Education institutions, schools and teachers should take proactive measures to enhance both their school attitudes and resilience.

First, education institutions should give high priority to returned migrant children in their daily management. Schools should establish a systematic approach to track the movement of these children, documenting essential information such as the time of their return, the identity of their guardians, academic performance in the inflow areas, and the specific academic challenges they encounter upon returning. This data can be used to tailor interventions and ensure a smoother transition into the local school system.

Second, schools should organize integration activities designed to help returned migrant children feel welcomed and connected. Activities like cultural and sports events, essay-writing contests, campus tours, and presentations about the school history can help these students develop a sense of belonging. Many respondents reported that they made friends and adjusted to their new schools through sports and group activities.

Third, it is essential for educational institutions to support faculty members in creating bridging materials, developing school-based curricula and conducting professional development seminars aimed at improving pedagogical strategies for teaching returned migrant children. Given that these children come from diverse educational backgrounds and have faced varying academic challenges, teachers should be trained to recognize the unique needs of these students and use culturally sensitive, inclusive teaching techniques. Through personalized counseling and targeted support, educators can help them to fill these gaps and ensure that returned migrant children have the tools they need to succeed academically. Additionally, performance evaluations for educators should include criteria related to the care and support of returned migrant children, encouraging educators to prioritize their needs.

Lastly, schools should implement programs designed to enhance the resilience of returned migrant children. Group counseling, psychological workshops, anti-stress and self-regulation training can provide essential emotional support, helping them cope with the stressors they face in adapting to a new academic environment. Such programs can promote resilience by helping students build coping mechanisms, foster emotional self-regulation, and strengthen their academic self-efficacy.

## 5 Conclusion

In conclusion, these findings suggest that the teacher-student relationship plays a crucial role in the academic adjustment of returned migrant children. A positive and harmonious teacher-student relationship is directly associated with improved academic adjustment. Additionally, this relationship’s effect is medicated by school attitude and resilience, which act as chain mediators. These findings highlight the importance of fostering strong teacher-student bonds and addressing both emotional and academic needs to facilitate the successful adjustment of returned migrant children to their new educational environments.

## Data Availability

The raw data supporting the conclusions of this article will be made available by the authors without undue reservation.
